# Designing Communication Feedback Systems To Reduce Healthcare Providers’ Implicit Biases In Patient Encounters

**DOI:** 10.1145/3613904.3642756

**Published:** 2024-05-11

**Authors:** Emily Bascom, Reggie Casanova-Perez, Kelly Tobar, Manas Satish Bedmutha, Harshini Ramaswamy, Wanda Pratt, Janice Sabin, Brian Wood, Nadir Weibel, Andrea Hartzler

**Affiliations:** Information School, University of Washington, Seattle, Washington, USA; Biomedical Informatics and Medical Education, University of Washington, Seattle, Washington, USA; University of California, San Diego, San Diego, California, USA; University of California, San Diego, San Diego, California, USA; University of California, San Diego, San Diego, California, USA; Information School, University of Washington, Seattle, Washington, USA; Biomedical Informatics and Medical Education, University of Washington, Seattle, Washington, USA; University of Washington, Seattle, Washington, USA; Computer Science and Engineering & Design Lab, University of California, San Diego, San Diego, California, USA; Biomedical Informatics and Medical Education, University of Washington, Seattle, Washington, USA

**Keywords:** Communication Feedback, Imlicit Bias, Healthcare, Healthcare Providers

## Abstract

Healthcare providers’ implicit bias, based on patients’ physical characteristics and perceived identities, negatively impacts healthcare access, care quality, and outcomes. Feedback tools are needed to help providers identify and learn from their biases. To incorporate providers’ perspectives on the most effective ways to present such feedback, we conducted semi-structured design critique sessions with 24 primary care providers. We found that providers seek feedback designed with transparent metrics indicating the quality of their communication with a patient and trends in communication patterns across visits. Based on these metrics and trends, providers want this feedback presented in a dashboard paired with actionable, personalized tips about how to improve their communication behaviors. Our study provides new insights for interactive systems to help mitigate the impact of implicit biases in patient-provider communication. New systems that build upon these insights could support providers in making healthcare more equitable, particularly for patients from marginalized communities.

## 1 INTRODUCTION

Implicit bias, based on a person’s gender, race, ethnicity, socioeconomic status, weight, height, and other aspects of a person’s physical characteristics and perceived identity can lead to health inequities [20, 28, 31, 43, 61]. Although often unintentional, healthcare providers’ implicit bias is associated with poor patient-provider communication, reduced patient satisfaction, and lack of patient trust in providers [11, 59, 60], which can negatively impact healthcare access, care quality, and inequitable outcomes [19, 48, 55]. Healthcare inequities associated with implicit bias are most visible in patients from marginalized communities. Discrimination and biases toward Black, Indigenous, and People of Color (BIPOC) [2, 12, 28, 43, 60] and lesbian, gay, bisexual, transgender, and queer/questioning (LGBTQ+) [6, 9, 17, 23, 24, 34, 39, 40, 60] patients are widely documented, motivating providers and healthcare institutions to implement educational support and tools to mitigate disparities resulting from these biases. Despite the growth of implicit bias recognition and management curricula and training interventions to address health disparities and advance equity [27], approaches to implicit bias instruction have mixed results [3, 63]. Bringing awareness to one’s implicit bias alone can result in dissonance, discomfort, and defensiveness [62, 64, 65, 72], thus, interventions that raise awareness of implicit bias should be paired with strategies that help individuals to understand and reduce its impact on patient care [63, 64]. While pairing awareness of one’s implicit bias with bias-mitigating strategies shows promise, many current provider education curricula that utilize this approach demonstrate varied success [72]. This mixed evidence suggests further exploration is needed to find alternative ways to target implicit bias recognition and management training.

Effective communication between patients and providers is essential to equitable care and may provide an opportunity through which artificial intelligence, or other advanced computing methods, can be applied to help reduce the impact of implicit bias. Poor communication between patients and providers has been associated with an increased risk of preventable medical errors and adverse events [4, 16, 53], and contributes to poor patient-provider interactions, reduced ratings of care [11], less patient satisfaction, and reduced trust in providers [30]. Racial and ethnic disparities in the quality of patient-provider communication have been widely documented, with Non-Hispanic White people experiencing the most effective patient-provider communication, compared to poorer communication experienced by BIPOC patients [10, 36, 50, 69]. In fact, provider implicit race bias has been linked with differences in their nonverbal communication patterns, including greater verbal dominance (i.e., conversational control) and slower speech with Black patients compared with White patients [11]. The disparities found in patient-provider nonverbal communication suggest that focusing on communication feedback tools to support providers in communicating more effectively with people from marginalized communities may provide a means for reducing the impact of implicit bias in healthcare [30].

Based on interviews conducted in prior work with primary care providers, the types of preferred tools to deliver communication feedback can be categorized as digital nudges, guided reflection, and data-driven feedback [14]. Providers’ perspectives on the design of these tools are necessary for effectively implementing them into clinical training and practice to ensure their usefulness, accessibility, and actionability while ensuring they are not disruptive or intrusive to clinical work. Gathering provider perspectives on the design of this technology will help uncover and address barriers to implementing these tools to raise providers’ awareness of the potential for implicit bias to shape patient interactions and manage the impact of implicit bias during healthcare interactions. Through 24 semi-structured design critique sessions with primary-care providers, divided across four iterative rounds, characterize providers’ preferences about how communication feedback tools should be designed and integrated into healthcare. We identified design goals to help providers meaningfully engage and make actionable changes in their communication practices to reduce the impact of implicit bias in patient-provider communication.

## 2 RELATED WORK

### 2.1 Feedback Tools For Healthcare Providers

Feedback tools that show promise in improving patient-provider communication are divided into (1) Feedback reports, (2) simulated patient interactions (e.g., through interactions with standardized patients), and (3) sensing technology to produce feedback from social signal processing (SSP) of real-time patient interactions.

#### 2.1.1 Feedback Reports.

Feedback reports refer to the monitoring and reporting of key indicators used to assess the care providers deliver to their patients [44]. These reports are generally created using data from medical records, registries, administrative or claims databases, observations, and/or patient surveys and designed to evaluate and motivate efforts to improve patient care [44]. Examples range from the use of electronic medical record data for reporting clinical quality measures among value-based primary care practices [8] to individual feedback to clinicians on their diagnostic performance [45] or prescribing patterns compared with peers [46]. In some instances, these reports can address providers’ communication by reporting their patients’ satisfaction with the care they received; this data is usually collected through surveys distributed to patients, such as the Consumer Assessment of Healthcare Providers & Systems (CAHPS) surveys [22]. Feedback reports are often not presented to providers until months after the data is collected and fail to provide actionable feedback to providers as the questions are typically close-ended and are not contextualized with what happened during the visit [71]. Furthermore, through an analysis of Medicare patients at risk of cardiac surgery, Dranove et al. identified unintended consequences of “report cards” that may incentivize providers and hospitals to decline to treat severely ill patients (i.e., to prevent the likely negative impact from report cards), leading to worse health outcomes for sicker patients [15]. Comprehensive guidelines for improving the efficacy of provider feedback reports have been published [44, 71]; however, these recommendations continue to be underutilized. Our findings support the need for engaging healthcare providers in design thinking to guide more timely, personalized communication feedback that helps providers deliver better care to patients from various backgrounds with all health statuses.

#### 2.1.2 Simulated Patient Interactions.

Leveraging simulated patient interactions with “standardized patients” (i.e., trained actors who are taught to portray a patient in a clinical scenario in a standardized way to make the educational experience consistent) [21], could be an impactful way of improving provider communication with patients. For example, standardized patients have been used to improve the knowledge and confidence of providers when raising the topic of organ donation with family members [54]. Other researchers have used computers to simulate patient interactions for providers to practice clinical communication during training. This approach has been applied in a variety of settings, such as using virtual reality (VR) to help medical students develop communication skills for engaging in challenging conversations [38]. More recent research has explored the application of simulated patient interactions in medical education to mitigate implicit bias in patient-provider interactions [35, 66]. Tija et al. report on the use of simulation-based learning with standardized patients in the development of an implicit bias recognition and management training programs [63] to mitigate clinician implicit bias [66]. implicit bias recognition and management refers to medical curricula designed to promote conscious awareness of biases and foster behavioral change [63]. Example implicit bias recognition and management strategies range from awareness raised through video observation of exemplar-biased patient interactions, self-reflection, and discussion about personal experiences with implicit bias, to skills acquisition through role play and simulation. By engaging patients from community-based organizations with a trusted and long-standing history of academic collaboration, Tija and colleagues [66] found their implicit bias recognition and management program provided clinical trainees with content highly tailored to the experience of local patient populations by focusing on identity, race, ethnicity, and local health system challenges related to structural racism [66]. Kanter et al. [35] engaged Black standardized patients in clinical workshops to decrease providers’ likelihood of expressing biases and negative stereotypes during interactions with patients of color during racially charged moments. They found that providers who engaged with the intervention had improved emotional rapport, responsiveness, and self-reported working alliances and closeness with Black standardized patients. Further research is needed to explore the efficacy of simulated patient interactions with other feedback tools. Innovative VR technology, such as MPathic-VR system, has been shown to improve awareness of nonverbal behavior and enhance communication skills by having providers practice how they communicate with patients through interacting with a virtual avatar [29, 38]. By targeting communication behaviors associated with implicit bias, such technology-based implicit bias recognition and management systems show promise for mitigating the impact of implicit bias in patient-provider interactions [30].

#### 2.1.3 Social Signal Processing.

Researchers have also investigated computational sensing technology to provide feedback to providers about their communication with patients, including social signal processing [5, 18, 33, 41, 51, 58, 68]. This research has centered around automated detection of verbal and nonverbal communication features from audio and video streams, such as how the words are spoken (e.g., tone of voice), the body language of those interacting (e.g., body movement, posture, and facial expression), and characteristics of the conversation (e.g., talk time, interruptions, sentiment). These communication features have been leveraged in tools to provide real-time feedback during the visit [18, 33, 51] and reflective feedback to providers after the visit [41, 58]. While these works are foundational research for describing interactions between patients and providers, none of these studies focus on the applications of this technology in connection to implicit bias. Gathering data about disparities in communication features shown to signal implicit biases (e.g., conversational control, speech rate) in connection to patient outcomes can help shed light on how communication feedback should be focused to decrease the impact of implicit patient-provider communication biases. Based on this existing research, there is an opportunity to leverage sensing technology to inform the content of the personalized communication feedback dashboards and educational resources to help mitigate the impact of implicit bias in patient-provider communication.

### 2.2 Provider Perspectives on Communication Feedback Tools

Few studies investigate healthcare providers’ perspectives regarding the design of communication feedback [44]. In a systematic review of the literature investigating the online delivery of evidence-based medicine to providers through digital tools, Opel found that providers’ needs for information seeking are not considered when designing online content for provider education [49]. Payne et al. found timely, personalized feedback that includes patient-level data on areas for improvement to be most acceptable to primary care providers; however, this feedback can generate resentment and negative emotions that impact its acceptability [52]. Loomis and Montague identified through design workshops with clinicians that specific, immediate feedback is important to and welcomed by clinicians; however, certain types of feedback (e.g., patient surveys) were viewed with skepticism and negatively impacted perceptions of utility and trust [42]. Dirks et al. identified that providers’ preferred modalities for tools to improve communication and raise awareness of implicit bias were digital nudges, guided reflections, and data-driven feedback [14]. We leveraged this existing literature to develop wireframes to guide conversations about providers’ preferences on design features for tools that provide feedback on implicit patient-provider communication biases.

## 3. METHODS

### 3.1 Study Design

We conducted four rounds of iterative, semi-structured design critique sessions with 24 primary care providers (6 providers per round) to refine communication feedback wireframes and align with provider preferences by answering the research question:

**RQ:** What are primary care providers’ preferences on design features for tools that provide feedback on implicit patient-provider communication biases during clinical interactions?

Study procedures were reviewed by the first author’s university’s Institutional Review Board and determined exempt by the first author’s university’s Institutional Review Board. Participants were recruited in the United States via convenience and snowball sampling through our Clinical Champions, who are a group of clinicians that serve in an advisory role to the research project and assist with recruitment and study design.

### 3.2 Wireframes

Based on prior work [14, 42], we developed three types of wireframes to show participants during design critique sessions. Through design interviews with primary care providers, Dirks et al. [14] identified three concrete modalities participants recommended for delivering communication feedback that raises awareness of implicit bias through digital tools: data-driven feedback, digital nudges, and guided reflections. Leveraging this related work, we created the wireframes used in this study, which illustrate Dirks et al.’s three design concepts for feedback on patient-provider communication biases, which were framed to support provider growth and had different timings for which they would be used as suggested: (1) *Data-Driven Feedback* (e.g., via traditional dashboards), (2) *Real-Time Digital Nudge* (e.g., alerts and ambient feedback during clinical interactions), and (3) *Guided Reflection* (e.g., via conversational agents and human mediation). We also incorporated the design considerations of Loomis and Montague [42]: feedback should be presented to support provider growth (e.g., the use of feedback and reflection as opposed to penalization), the timing of critical feedback is important to consider, and feedback should be integrated into short- and long-term provider workflows (e.g., the wireframes provide feedback in different modalities at different stages in a providers workflow).

#### 3.2.1 Data-Driven Feedback.

**Figure 1:**
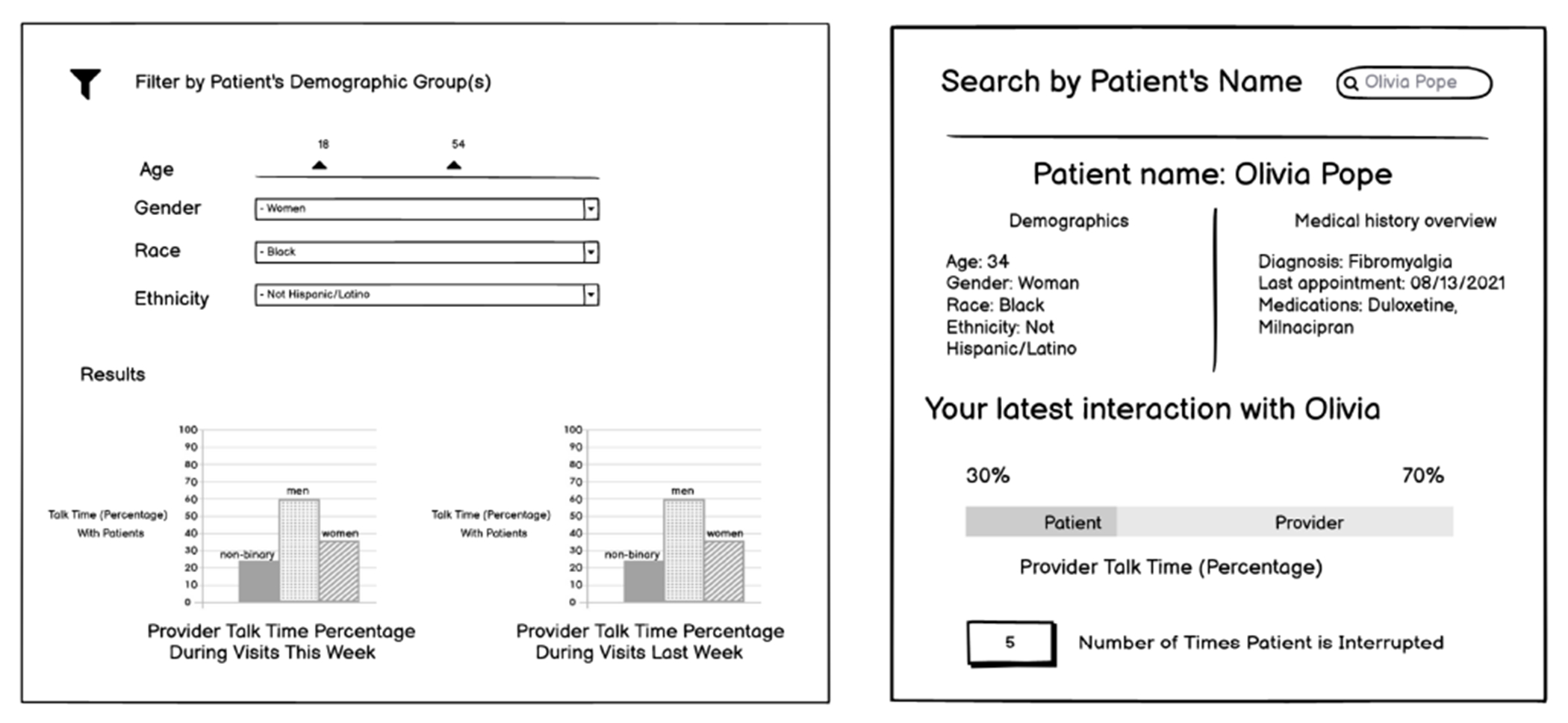
Round 1 Data-Driven Feedback Wireframes - Multi-Patient View (left) and Patient-Specific View (right)

The purpose of data-driven feedback is to give providers insight into their past interactions with patients after several visits, grounded in quantitative communication metrics to identify behavior patterns with patients from different demographic backgrounds (*See* Figure 1). As dashboards have high glance-ability, they allow providers to see a graphical representation of how their interactions went with specific patient groups and filter to the specific demographics or characteristics of interest. We divided our data-driven dashboard into two views: a multi-patient view to represent how the provider interacted with groups of patients and a patient-specific view to represent how the provider interacted with an individual patient they could search by name.

#### 3.2.2 Real-Time Digital Nudge.

**Figure 2:**
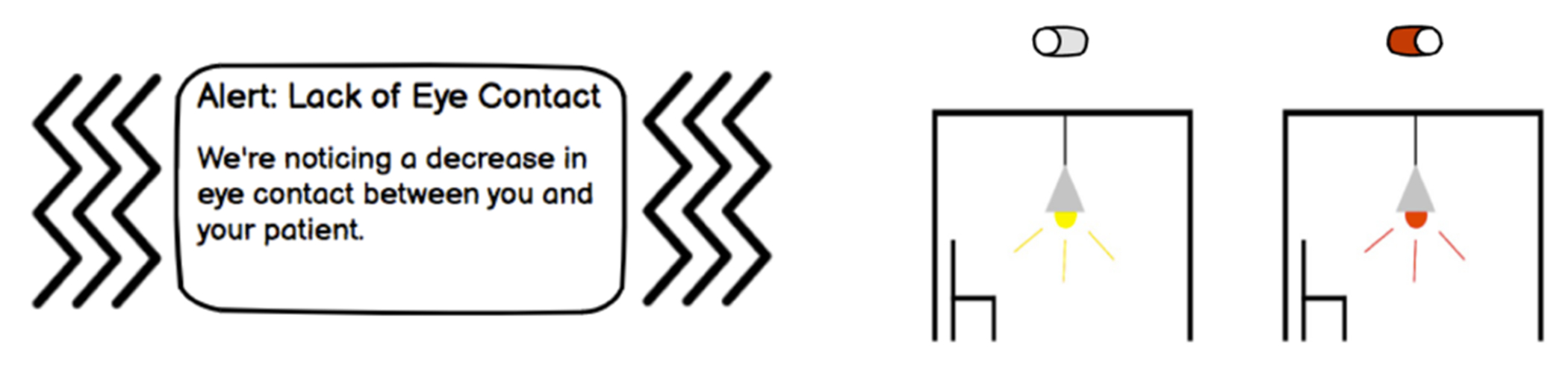
Round 1 Digitial Nudge Wireframes - Smartwatch notification (left) and exam room lighting change (right)

The real-time digital nudge aims to raise providers’ awareness of opportunities to change certain non-verbal behaviors that may improve their current interaction with a patient during a visit (*See* Figure 2). This nudge can be represented in different ways, such as through various images, texts, and notifications on devices in the exam room during a patient visit; for example, through smart-watch alerts or lighting changes in the exam room.

#### 3.2.3 Guided Reflection.

**Figure 3:**
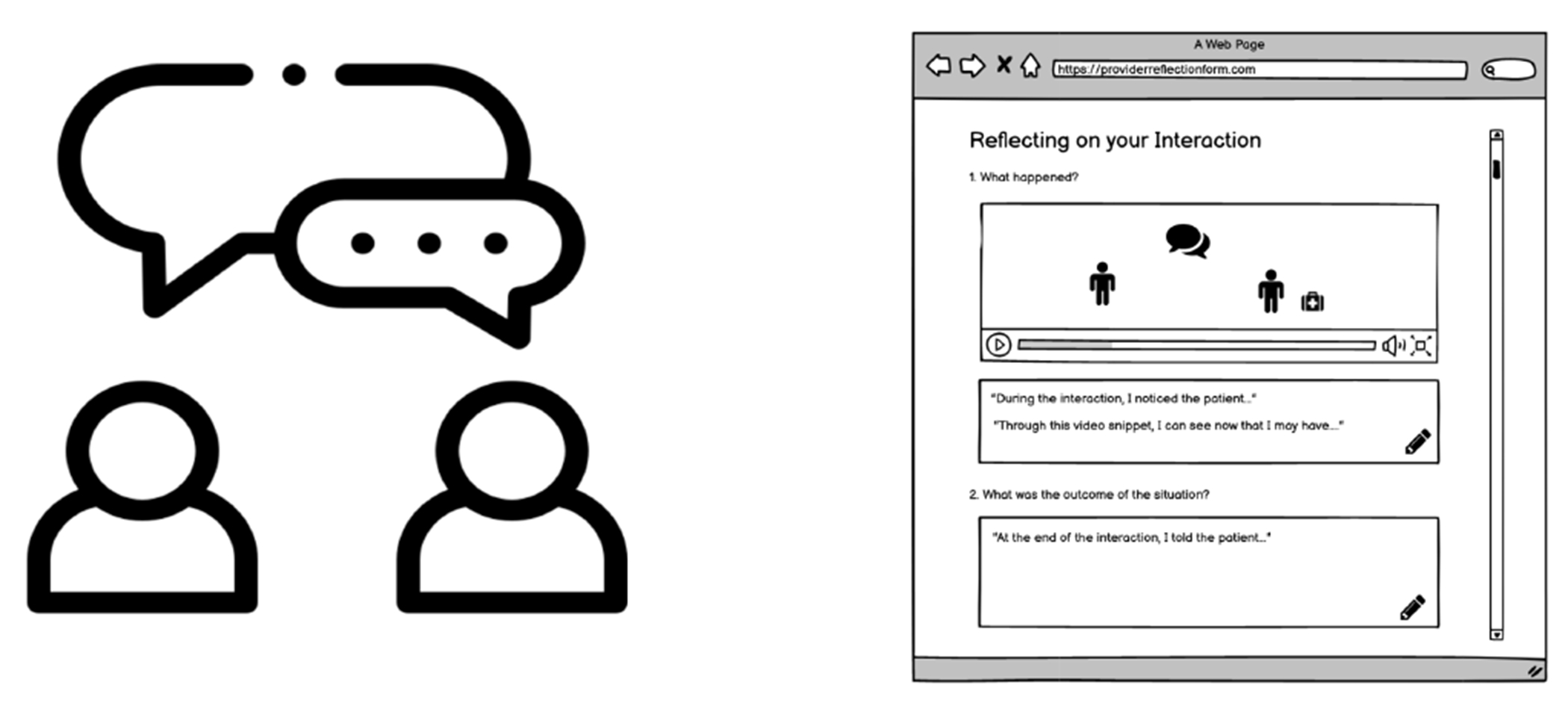
Round 1 Guided Reflection Wireframes - In-person debrief (left) and Gibbs reflective cycle (right)

The purpose of the guided reflection is to help providers strategize on how to improve their behavior with patients and consider how their behaviors may impact interactions with patients to be more supportive of their patients’ needs (*See* Figure 3). The guided reflection tool was based on the Gibb’s reflective cycle, which is a six-stage reflective teaching model that provides a structure for examining and learning from repeated experiences [25] and is commonly used with nursing students [56, 70]. This reflection was designed to be used outside the patients’ visit when the provider can develop a plan to improve their interactions with patients.

These three design concepts focus on presenting non-verbal communication features between patients and providers during clinical interactions, such as body language, eye contact, posture, and speech patterns (e.g., talk time and interruptions). All the wireframes were fabricated with fictional data that was inspired by but did not present real interactions between patients and providers. Wireframes were initially low-fidelity to invite feedback and encourage participants to concentrate on the communication features instead of aesthetic details and were later increased to medium-fidelity to see if and how participant critiques would change. Between rounds, we used affinity diagramming [32] to iterate on the wireframes by incorporating participant feedback.

### 3.3 Data Collection

We collected data through an online survey and remote individual design critique sessions conducted via Zoom to accommodate participants’ busy schedules and improve the geographic reach of the study participation.

The *online survey* collected information about demographics (i.e., gender, race, ethnicity, self-selected identity), clinical practice experience (i.e., clinical role, years in role, panel size), and two published measures, including the day-to-day unfair treatment subscale of the Experience of Discrimination (EOD) measure [37], and a measure of bias awareness [26].

Each *design critique session* was administered by two interviewers, one to lead the session by asking participants questions and another to take notes during the session. For those notes, the second interviewer filled out a template that followed the flow of the interview questions for analysis. Sessions were video recorded and conducted individually with each provider, lasting between 45 and 60 minutes. Each session had two parts, spanning five key concepts (i.e., general use, context of use, features, actionability, and institutional and personal barriers) which were inspired by the themes presented by Dirks et al. [14] and Loomis and Montague [42].

Part 1 consisted of presenting wireframes to participants and asking them for feedback on the features of each wireframe by describing how they envision the wireframes’ general use, context of use, and desired features. Throughout the rounds, we presented different types of communication cues and visualizations to find the most valuable cues for providers to depict their interactions with patients. Questions asked in Part 1 of the session were:

- **General Use:** How would you imagine using this wireframe? What works or does not work for you?
- **Context of Use:** When and where would you envision using this wireframe?
- **Features:** What is the most important information you would want from this wireframe?

Part 2 consisted of collecting participant thoughts about the implications of implementing tools like the ones represented in the wireframes in clinical practice, focusing on how to make the feedback provided actionable and any anticipated personal or institutional barriers. Questions asked in Part 2 of the interview were:

- **Actionability:** After receiving feedback on communication from any one of these wireframes, what might you need to make that feedback actionable?
- **Context of Use:** What personal and/or institutional barriers would you anticipate when using the kinds of wireframes we shared with you today?

This protocol was pilot-tested and refined with three researchers not involved in the study design.

### 3.4 Analysis

#### Online survey:

We summarized survey data with descriptive statistics to characterize the participant sample. We report the mean and standard deviation of the 6-point agreement ratings for each item of the Bias Awareness measure [26]. We followed the published rubric for scoring the EOD measure from 0 to 50, with higher scores reflecting more frequent experiences of discrimination in day-to-day life [37].

#### Design critique session:

We iteratively analyzed session data using thematic analysis. After each round was completed, one research team member compiled participant feedback collected in the note-taking template into a Miro board [7] for collaborative, deductive thematic analysis. The board contained a picture of each wireframe presented to the participants for a given round, sticky notes with participant quotes about each prototype (Part 1), and implications of prototype use (Part 2). Using this board, research team members (initials redacted for blind review) conducted a two-hour remote synchronous affinity diagramming session [32]. This process involved team members individually reviewing the feedback for a wireframe, documenting themes that emerged to them, and then coming together to discuss themes until a consensus was reached about the most important takeaways from participants’ input for every wireframe to inform the changes needed for the next round. At the end of each round, themes were organized by wireframe (i.e., general use, context of use, and important features), suggestions for how to make communication feedback actionable, and anticipated institutional and personal barriers for using communication feedback tools.

## 4 RESULTS

We completed four rounds of design sessions with a total of 24 participants, which included six new participants each round, P01-P24 (*See* Table 1). The majority of participants were White, Non-Hispanic, or Latino/a/x/e, and were evenly split between women and men. Five participants self-identified as BIPOC, three as LGBTQ+, and two as LATINX. Most participants were medical doctors, but two participants were nurse practitioners, and one was a doctor of osteopathic medicine. Participants had 1.5 to 42 years of experience. Furthermore, participants had an average Experiences of Discrimination score [37] of 17.5 out of 50 possible (SD = 15.6, range = 0 - 47.5), and generally agreed that they are objective in their decision-making and that people of different social groups are treated differently and do not have equal opportunities for achievement [26] (*See* Table 2). Wireframe content and fidelity were improved in each round based on findings from the previous round of participant critique; interestingly, participant insights about general use, context of use, and features from Part 1 and actionability needs and anticipated barriers from Part 2 were thematically consistent across all rounds. In this section, we first describe the feedback provided for each wireframe and the iterative evolution of each design (Part 1 Results), then describe participant recommendations for making communication feedback actionable and the institutional and personal barriers participants may face when implementing communication feedback tools (Part 2 Results).

### 4.1 Part 1 Results – Wireframe Evolution Based on Participant Feedback

#### 4.1.1 Data-Driven Feedback.

**Figure 4:**
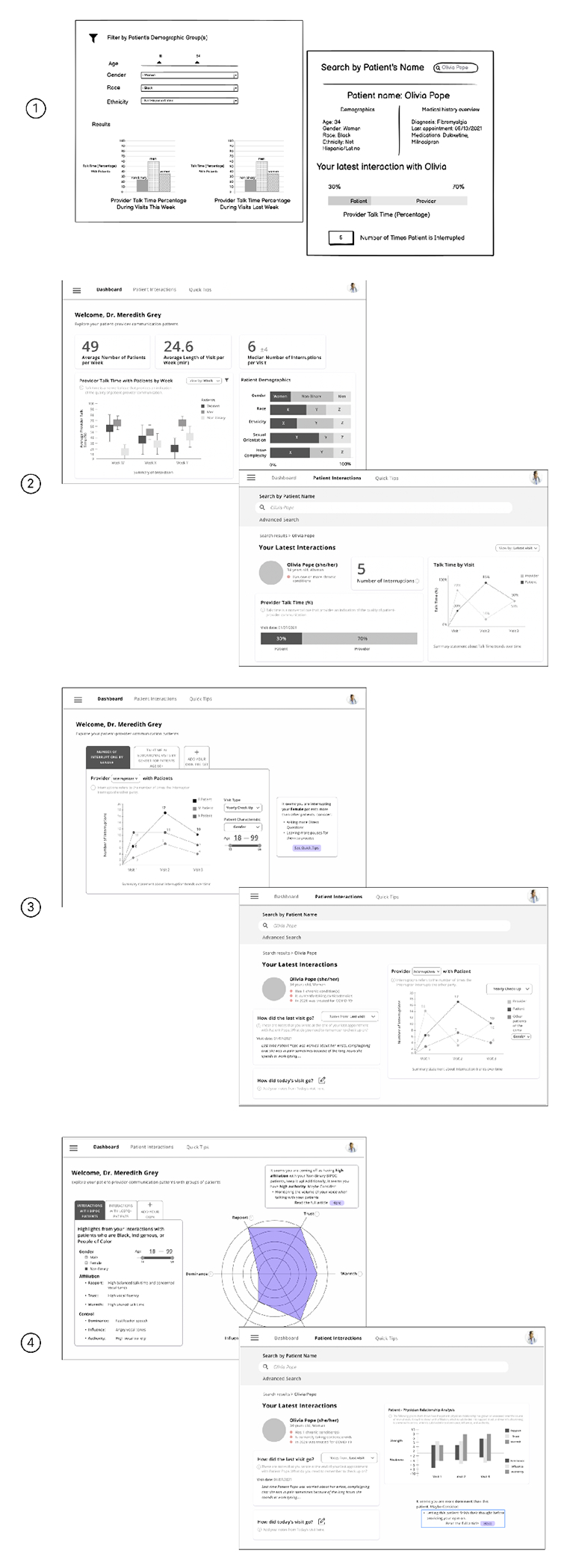
This figure shows how the data-driven feedback wireframe changed for each round. Each wireframe used fabricated data and served as a tool to guide the session.

Figure 4 shows the wireframe evolution across the four rounds of design critique. After conducting four rounds of design critique sessions, the Data-Driven Feedback wireframe maintained two distinct views; (1) a “multi-patient” view where providers see information about the communication patterns across all of the patients they care for, and (2) a “patient-specific” view where providers can see in-depth communication patterns with a specific patient across that patient’s visits. Nearly all participants preferred the data-drive feedback wireframe over the Real Time Digital Nudge and Guided Reflection wireframes.

Across sessions, fourteen of the 24 participants (58%) prioritized comparisons of various patient demographics throughout the data-driven feedback wireframes. For instance, P10 explained that the information presented could be illuminating as it *“might show that there is some disparity and I am interrupting one group over another*.” P10 also highlighted these dashboards may help them adjust their perspective from *“[I am] having less luck on counseling [a certain group of patients] on [a certain] disease*” to a place where they recognize “*it has less to do with [the certain group of patients] but the way I relate to them*.” Beyond patient demographics, 9 of 24 participants (37.5%) also wanted to see comparisons and changes regarding their communication behavior over time, with P05 elaborating that “*these habits [of adjusting my communication with patients] take a while to sink in*.”

Regarding the communication features presented in these dashboards (e.g., provider talk time, number of interruptions), 16 of the 24 (67%) participants wanted evidence on why the presented features are the most meaningful and accurate for depicting the nature of the interaction with the patient. P02 explains, *“not all interruptions are bad… the reality of the visit is that I’ve got 15 minutes… but sometimes [interruptions] are what you do to get the information*.” These participants felt that the values of the communication features presented alone lacked the context they needed to be meaningful and actionable. Furthermore, these participants sought more information about the ideal or desirable metrics for these communication features.

Participants across rounds also described differences in cadences for which they would want to review the patient-specific and multi-patient data-driven feedback; however, most did not want to receive feedback during their visit while the patient was in the room. Seventeen of 24 participants (71%) mentioned how feedback during the visit could be distracting, overwhelming, and confusing, with P20 stating that “*to try and recognize [a certain behavior] in real-time and make an adjustment – that’s tough*.” Eleven of 24 participants (46%) explicitly mentioned envisioning themselves reviewing the dashboards before or after a visit, with P14 explaining how “*looking at this before a patient visit may be helpful to frame behavior* going into an interaction.

#### 4.1.2 Real-Time Digital Nudge.

**Figure 5:**
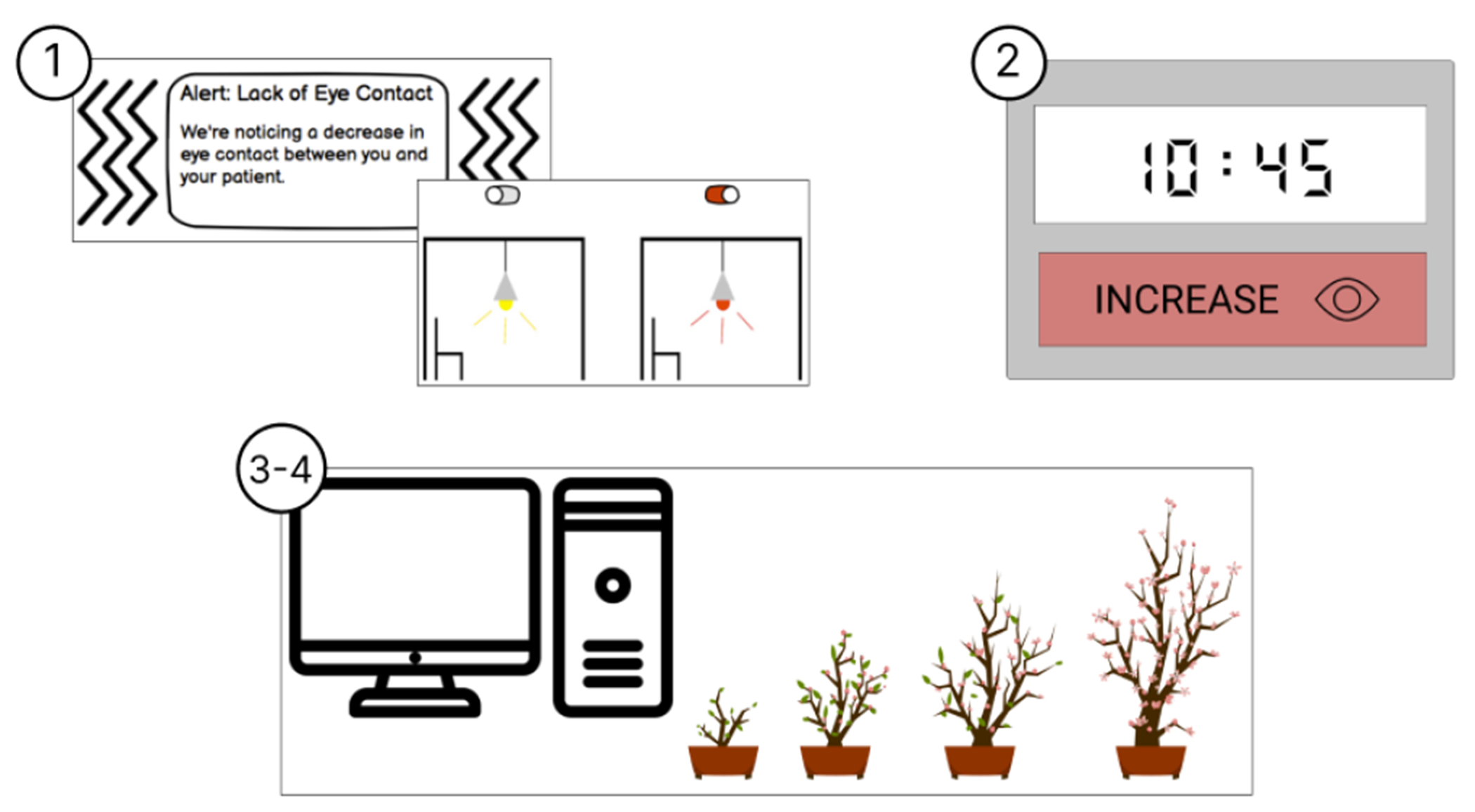
This figure shows how the real-time digital nudged changed across rounds from across rounds. Round 1 wireframe includes a smartwatch notification and a change in ambient lighting (top left), Round 2 featured a wireframe that was a smart clock that would prompt providers to improve non-verbal communication cues (top right), and Round 3 and 4 featured a tree that would grow and blossom if both the patient and provider’s non-verbal communication behavior was indicating that the conversation was going well (bottom middle)

Figure 5 shows the evolution of the real time digital nudge wireframe across the 4 rounds of design critique. The Real-Time Digital Nudge wireframe was designed to inform providers about their real-time communication behaviors during their patient encounters. This wireframe was depicted in various modalities, including smartwatch notifications, ambient light changes, and a digital picture frame with a tree that would grow and blossom. Overall, this wireframe elicited a variety of impressions and was least preferred compared to the Data-Driven Feedback and Guided Refection wireframes. The final iteration of the Real-Time Digital Nudge emphasized highlighting what providers were doing well during an interaction to encourage more desirable communication behavior and reduce the potential implications of a patient noticing the intervention, received the most positive impressions from participants.

While four participants (17%) had positive impressions regarding the general use of the digital nudge, stating that the tool is a “*nice encouragement of good behavior*” (P14), the remaining 20 participants (83%) expressed much hesitancy about implementing a feedback tool like this in exam rooms during patient visits. The main concern for these participants was the potential for the digital nudge to be distracting to both the patient and the provider. P20 explained, “*[feedback] in real-time is really tough – there’s a lot going on in a visit and both the provider and the patients have a lot to address which may make it difficult to pay attention to [the digital nudge] in a meaningful way*.” P05 elaborated, stating:

“*It feels counter-intuitive and somewhat ironic that you’re telling somebody to look away from the patient at their watch to tell them to look at the patient more*.” – P05

As an alternative solution, P14 and P16 expressed being interested in having their communication behavior shown to them after a patient visit or at the end of the day, rather than in real-time during the visit; for example, P14 remarked that it would be nice to have a summary at the end of the day describing “*you had really good [interactions] because you did x, y, and z, things with your body*.”

#### 4.1.3 Guided Reflection.

**Figure 6:**
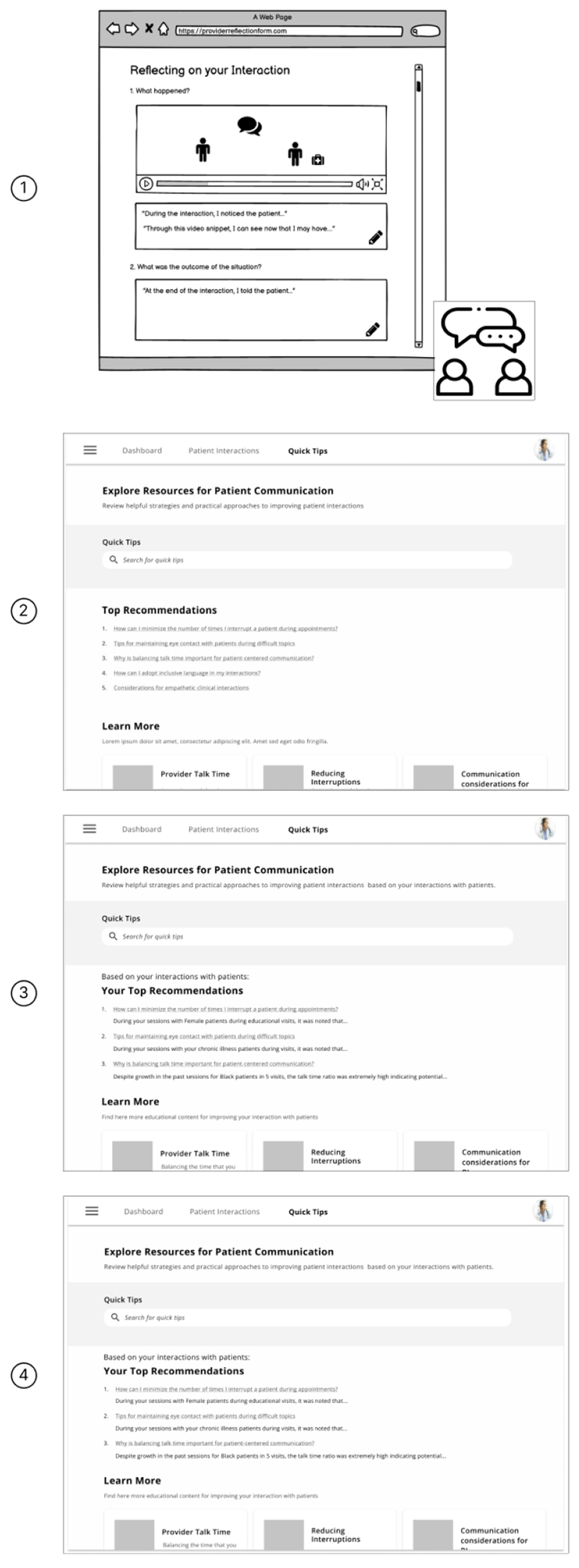
This figure shows how the guided reflection wireframe changed from Round 1, which featured an in-person debrief and Gibbs reflective cycle (top), and Rounds 2-4, which featured a Quick Tips page that had actionable suggestions that were tied directly to the provider’s communication behavior. The data in these wireframes was fabricated and used to guide the session.

Figure 6 shows the evolution of the guided reflection wireframe across the 4 rounds of design critique. Our initial Guided Reflection wireframes depicted in-person or chatbot debriefs by reflecting on video snippets of patient interactions that could be improved. Participants in the first round of design critique sessions immediately pushed back on this wireframe, explaining that while seeing video snippets of their interaction would be helpful, frequent guided debriefs would be onerous and “*too much of a chore*” (P04), with little to no advice about improving their interaction. Additionally, these participants explained that this type of review would be too time-consuming. To address this, the wireframe evolved across the 4 rounds to be a “Quick Tips” page where, based on trends in the provider’s communication behavior, they would be shown five tips or things to think about with the option to learn more by reading an academic paper or another resource.

Seventeen of 24 participants (71%) described wanting tips and recommendations personalized to their specific behavior with patients. P23 explains providers “*don’t want to spend a lot of time doing stuff that [they’re] already doing pretty well*” and “*want to focus on the stuff that’s going to be high yield*.” Eight of 24 participants (33%) also viewed the Quick Tips page as a valuable educational resource, envisioning the feedback they received from the data-driven dashboards being paired with information in the Quick Tips page to serve as a way to debrief and determine opportunities for improvement; six participants (25%) were interested in seeing ‘deep dives’ on patient demographics:

“*If you find that you have a subset of patients that it’s consistent where you’re scoring lower then maybe you could then kind of look at some of the characteristics that happened during those times and you might be able to do some pre-visit planning that’s a little bit different… But how can– how can I readjust to improve things there? And this might be where coaching or some kind of an educational thing can help out here*.” – P22

Four of 24 (17%) providers specifically mentioned incorporating the Quick Tips page into their continuing education requirements, with one provider mentioning its use as a potential way to receive credit for diversity, equity, and inclusion training (P15).

Seventeen of 24 participants (71%) worried about having the time and/or institutional support to engage with the Guided Reflection tool; however, some participants suggested workarounds for this:

“*I would love something like if we if you were going to assess me and then there’s I get this little thing in my e-mail, then here are some resources. So I would love, like, a button I could click on after I see my data. And then or even a video saying course or I mean an assigned course is OK, you know, through video. But even if there’s like here are some resources click that we could read*.” – P13

Participants also found the Quick Tips page especially helpful for students or residents, who may have more time to review their feedback. As P18 stated, “*I could see this [as] useful during [the] professional development of young physicians. If I were in practice, this is too busy. But in an educational setting, this would be useful*.”

### 4.2 Part 2 Results - Implications

#### 4.2.1 Making Feedback Actionable.

Participants highlighted several ways in which they could imagine making the feedback provided by the wireframes actionable. Nineteen of the 24 participants (79.2%) wanted actionable, personalized feedback on improving their implicit communication bias. Examples participants described were succinct feedback, graphs, and tips for improving their behavior, information on what to do following receiving feedback, supporting educational materials on why certain communication behaviors matter, and having the informal opportunity to choose to discuss the feedback they received with other providers or trained professionals in a non-punitive way. Participants largely focused on the quick tips resources, wanting to see support through courses, “communication coaches” (i.e., personal communication coaching by specialist consultations [1]), readings, podcasts, or videos that are presented in a way that encourages provider wellness and improvement. Furthermore, participants identified a clear, beneficial link between the data displayed in the data-driven feedback views and the quick tips page:

“*It’d be fine to have [the quick tips] in its own separate page like this but it could also be direct from the dashboard and results… so that while you’re paying attention to [the dashboard] you can follow [the dashboard] up [with the quick tips]*” – P08

Sixteen of the 24 participants (67%) wanted to compare metrics across in-depth patient demographics and have their behavior patterns with specific patients groups linked to actionable suggestions within the quick tips page for how to better communicate with those groups. Twelve of the 24 participants (50%) were also interested in seeing how their communication behavior changes over time to document their progress and provide motivation for continuous improvement. P22 elaborated on this concept while describing what kind of information would be helpful for them and their peers in their clinic to make actionable changes in their behavior:

“*Giving us information on how to better enact, react, interact with diverse patient populations is always a good thing. Because some people, it’s not that we don’t want to change. I think some just don’t know how to change and, you know, if there are resources readily available saying, ‘hey, this is an area of for improvement for you’ and then ‘here’s the resource on how to do that,’ I think that would be helpful*.” – P22

Furthermore, nine of the 24 (38%) participants were interested in being compared with other providers on “*institutional… and national benchmarks*” (P06). P06 continues, explaining:

“*I do not even know the composition of my patients[race-wise]… it would be nice to see percentages. I would like to understand if my comparison is different from white providers… are they going to have shorter visits with their patients of color vs my data then also understand if I am spending more time with these patients it is because they are presenting with more illnesses… comparing providers of color vs non-providers of color*” – P06

While the opportunity for actionability based on the data-driven feedback and quick tips wireframes were clear, 20 of the 24 participants (83%) anticipated the real-time digital nudge wireframes may inhibit their ability to meaningfully engage with their patients during visits by serving as a distraction to both patients and providers.

#### 4.2.2 Detailing Institutional & Personal Barriers.

Participants described institutional and personal barriers they anticipated may arise when implementing an implicit communication bias feedback tool. Twenty of the 24 participants (83%) were worried about having enough time to effectively engage with implicit communication bias tools during practice hours, especially if providers were not incentivized or compensated for this time. Concern was raised about whether or not institutions would recognize the importance of implicit patient-provider communication bias enough to pay for tools to be purchased and incorporated into the clinical workflow, as well as the security of the data collected by the tool and its compliance with HIPAA regulations. Three participants proposed that if the tool was integrated with the institution’s Electronic Health Record or if the tool was paired with an organization’s diversity, equity, and inclusion initiatives, it may help overcome some of these barriers. Participants explained that they also worried that the data collected by the tool could be used punitively by their supervisor or insurance companies based on how providers perform. Two participants (8%) also explained that they worried some providers might not believe that reducing their implicit bias is important enough to dedicate their time to it.

## 5 DISCUSSION

After conducting 24 semi-structured design critique sessions, we learned that participants preferred a feedback dashboard with actionable educational resources (e.g., “quick tips”) to review outside the context of patient visits. Consistent with prior literature [14, 52], participants emphasized their desire for personalized, timely, patient-level feedback on their communication behavior. They described implementation barriers to consider, including appropriate compensation and time to review feedback, and seamless integration into their clinical workflow, rejecting the potential distraction of real-time nudges explored in prior literature [14, 18, 33, 51]. Here, we expand upon existing literature surrounding providers’ preferences on how to design communication feedback tools [14, 42, 52], with a focus on historically marginalized patients, to refine these design preferences while integrating innovative computing methods [5, 18, 33, 41, 51, 58, 68]. Through this, we create a jumping off point for provider-facing, social signal processing-generated interfaces geared toward raising awareness of implicit bias that consider the design preferences of providers.

Implicit bias perpetuates healthcare inequities and manifests in nonverbal patient-provider communication, such as body language and vocal patterns like interruptions. For example, providers with stronger implicit race attitudes favoring White people versus Black people, express greater conversational dominance with Black patients compared with White patients [11]. Effective communication between patients and providers is essential for equitable care and provides an opportunity through which implicit bias can be addressed [30]. Advanced computing methods are well-poised to address implicit bias through systems that extract meaningful communication signals from patient-provider interactions. The participants in our study frequently questioned the communication features included in the wireframes (i.e., interruptions, talk-time, etc.), noting that many of the features required the context of the conversation to determine the “acceptable” level (i.e., whether the provider dominates the conversation to explain a diagnosis or has balanced talk time when asking the patient about their symptoms). A possible alternative is to illustrate “social signals” rather than more specific communication features. “Social signals” refer to observable behaviors displayed during social interactions, often expressed through nonverbal communication like turn-taking [67]. One approach to characterizing such socioemotional communication signals during medical encounters is the Roter Interaction Analysis System is a widely used, manually applied, coding scheme [57]. the Roter Interaction Analysis System has been shown to identify social signals associated with provider implicit biases in patient-provider interactions [11] and can be leveraged to integrate well-established ways of describing patient-centered communication in social signal processing systems.

Echoing through participants’ responses is a desire to be a better provider to all patients, but also an honest recognition that there are only so many hours in the workday. Many providers already feel stretched beyond their capacity, supported by high documented rates of burnout amount providers [13]. Many providers welcome feedback and find it important; however, feedback should be actionable, presented to support provider growth, allow for adequate processing of critical feedback, and be integrated naturally into provider workflows to not distract providers from their clinical work [42, 52]. Providers recognized that tools similar to the wireframes could be a great opportunity to learn more about how they communicate with patients; however, an additional tool or requirement in exam rooms and clinics is likely to elicit scrutiny from providers, especially if there is a potential for it to become punitive.

Rather than forcing a solution that may lead to further provider burnout, we suggest the integration of the tools and technology described here with the preexisting education requirements providers already must complete. Our findings describe a clear opportunity to enhance patient-centered communication curriculum with personalized feedback on how each provider communicates and the unique populations they serve, elevating the integration of implicit bias recognition and management, and other diversity, equity, or inclusion-focused initiatives into the materials providers consume. The emphasis on personalized data may help these educational materials extend beyond continuing education, providing an opportunity for ‘continuous’ education rather than ‘continuing’ education, where providers may be incentivized to review these materials more than what is required because of their uniquely personalized nature. These “Just-in-Time Adaptive Interventions” for patients have grown to provide personalized behavior change support [47]. We call for the exploration of personalized interactive systems for providers as a way to modernize communication training and educational requirements through the use of technology, such as social signal processing [11] and VR simulated patients [27].

Future work is needed to corroborate the best way of incorporating these wireframes into clinical training and educational contexts using high-fidelity prototypes that visualize real communication data between patients and providers. Additionally, research is needed to understand the implications and efficacy of putting the necessary social signal processing technology in exam rooms to capture the data necessary to populate accurate visualizations of patient-provider communication behavior; considering the needs, wants, and concerns of both patients and providers, particularly those from historically marginalized communities.

## 6 LIMITATIONS & FUTURE WORK

This study has a number of strengths as well as limitations. Although participants were recruited through convenience and snowball sampling, the participant sample demonstrates diversity with respect to gender and self-selected identity. However, the majority of participants were non-Hispanic White individuals, which could have resulted in them failing to raise barriers or ideas that providers who are part of marginalized racial and ethnic groups find important for receiving feedback about their communication. Furthermore, we did not collect the geographic area where participants practice, which may mean this study could lack varied cultural contexts of healthcare, which is a necessary addition to future research in this area. Future work should explore how the perceptions and design suggestions of participants from different demographic backgrounds, clinic types and sizes, and patient populations served might vary to provide a deeper contextual lens. This lens is essential for ensuring effective interventions that benefit both providers and patients from all backgrounds.

Additionally, the wireframes presented in this study used fabricated data that did not portray actual patient interactions with participating providers. This could contribute to the participants not finding the information as useful or overlooking important aspects of their design values as the figures are not based on their actual interactions. Future work should test how visualizing actual provider data in these wireframes impacts the design recommendations of providers.

## 7 CONCLUSION

Through 24 semi-structured design critique sessions with primary-care providers divided across four iterative rounds, we found that providers seek transparent metrics indicating the quality of their communication with a patient and the trends in communication patterns over time across detailed patient demographics. Based on these metrics and trends, providers want interactive systems that provide actionable, personalized feedback about how to improve their communication behaviors. These systems need to be integrated into clinical workflows and provide non-punitive educational support. Future research is needed on how to corroborate the application of social signals to generate communication feedback in educational settings. Designs based on insights from our study could help mitigate the impact of implicit patient-provider communication bias and support more equitable healthcare for patients from marginalized communities.

## Figures and Tables

**Table 1:** Participant demographics, including age, gender, race, ethnicity, and self-selected identity.

Age	
Mean (SD),	45 (11),
Range	31 - 68

Gender	
Woman	12 (50%)
Man	12 (50%)

Race	
White	16 (64%)
Black/African American	2 (8%)
Asian Indian	1 (4%)
Chinese	1 (4%)
Other Asian	1 (4%)
Other: “Mixed”	1 (4%)
Other: “Latina”	1 (4%)
Decline to State	2 (8%)

Ethnicity	
Hispanic / Latino/a/x/e	2 (8.3%)
Not Hispanic / Latino/a/x/e	20 (83.3%)
Decline to State/No Response	2 (8.3%)

Self-Selected Identity	
BIPOC: Black, Indigenous, and People Of Color	5 (20.8%)
LGBTQ+: Lesbian, Gay, Bisexual, Trans, Queer, and other identities	3 (12.5%)
LATINX	2 (8.3%)
None	13 (54.2%)
Decline to State	1 (4.2%)

**Table 2:** Participant clinician role, years in role, panel size, Experiences of Discrimination Scale score, and Bias Awareness Measure.

Clinician Role	
Nurse Practitioner (NP)	2 (8.3%)
Doctor of Osteopathic Medicine (DO)	1 (4.2%)
Medical Doctor (MD)	21 (87.5%)

Years in Role	
Mean (SD),	15.7 (12.1),
Range	1.5-42

Approximate Panel Size (Number of Patients)	
Mean (SD),	368.5 (457.3),
Range	0-2000

Experiences of Discrimination Measure (EOD) *	
EOD Measure Score - Mean (SD),	17.5 (15.6),
Range*	0-47.5

Bias Awareness Measure (1 “Strongly Agree” to 6 “Strongly Disagree”)	
Personal Bias	Mean (SD)

*In most situations, I am objective in my decision making*	2.23 (1.08)
*Biases do not usually influence my decision making*	3.17 (1.52)
*Gender identity affects the types of biases that people have against other people*	1.78 (1.14)

Societal Bias	Mean (SD)

*People in today’s society tend to treat people of different social groups (e.g., race gender, class) equally*	4.68 (1.69)
*Society has reached a point where all people, regardless of background, have equal opportunities for achievement*	5.65 (0.70)

Biases in Healthcare	Mean (SD)

*In health care, bias against others is no longer a problem in the area of hiring*	5.13 (1.26)
*In health care, bias against others is no longer a problem in the area of promotion*	5.17 (1.34)
*In health care, bias against others is no longer a problem in the area of leadership*	5.43 (0.97)

* (EOD scores range from 0 to 50, with higher scores representing more frequent experiences)

## CCS CONCEPTS

• Human-centered computing → Collaborative and social computing; Visualization systems and tools; • Applied computing → Health informatics.
